# Transcriptional and post-transcriptional regulation of the ionizing radiation response by ATM and p53

**DOI:** 10.1038/srep43598

**Published:** 2017-03-03

**Authors:** Ishwarya Venkata Narayanan, Michelle T. Paulsen, Karan Bedi, Nathan Berg, Emily A. Ljungman, Sofia Francia, Artur Veloso, Brian Magnuson, Fabrizio d’Adda di Fagagna, Thomas E. Wilson, Mats Ljungman

**Affiliations:** 1 Department of Radiation Oncology, University of Michigan Comprehensive Cancer Center, Translational Oncology Program and Center for RNA Biomedicine, University of Michigan, Ann Arbor, Michigan, USA; 2 IFOM - The FIRC Institute of Molecular Oncology Foundation, Milan, Italy; 3 Istituto di Genetica Molecolare, Consiglio Nazionale delle Ricerche, Pavia, Italy; 4 Novartis Institutes for Biomedical Research, Cambridge, Massachusetts, USA; 5 Departments of Pathology and Human Genetics, University of Michigan Medical School, Ann Arbor, Michigan, USA; 6 Department of Environmental Health Sciences, School of Public Health, University of Michigan, Ann Arbor, Michigan, USA

## Abstract

In response to ionizing radiation (IR), cells activate a DNA damage response (DDR) pathway to re-program gene expression. Previous studies using total cellular RNA analyses have shown that the stress kinase ATM and the transcription factor p53 are integral components required for induction of IR-induced gene expression. These studies did not distinguish between changes in RNA synthesis and RNA turnover and did not address the role of enhancer elements in DDR-mediated transcriptional regulation. To determine the contribution of synthesis and degradation of RNA and monitor the activity of enhancer elements following exposure to IR, we used the recently developed Bru-seq, BruChase-seq and BruUV-seq techniques. Our results show that ATM and p53 regulate both RNA synthesis and stability as well as enhancer element activity following exposure to IR. Importantly, many genes in the p53-signaling pathway were coordinately up-regulated by both increased synthesis and RNA stability while down-regulated genes were suppressed either by reduced synthesis or stability. Our study is the first of its kind that independently assessed the effects of ionizing radiation on transcription and post-transcriptional regulation in normal human cells.

Ionizing radiation causes acute cell injury by inducing damage to lipids, proteins and DNA by direct ionization of target molecules and indirectly via hydroxyl radicals formed by radiolysis of water molecules^1^,^2^,^3^. DNA damage inflicted by IR includes base damage and single- and double-strand breaks (SSBs and DSBs)^1^. DSBs are the most toxic lesions after IR and they are repaired primarily by non-homologous end-joining (NHEJ) and homologous recombination (HR)^4^,^5^. In addition to DNA repair, cells activate a DNA damage response (DDR) that induces cell cycle arrest or apoptosis to suppress the mutagenic effects of IR^6^,^7^. The major orchestrator of the DDR is the stress-response kinase ATM that phosphorylates more than 700 substrates following activation by IR^8^. One key substrate of ATM is the tumor suppressor p53 that acts as a transcription factor^9^,^10^,^11^. Phosphorylation of p53 by ATM allows p53 to accumulate and activate its sequence-specific DNA-binding activity promoting induction^12^ or repression^13^,^14^ of expression of target genes. Many of these target genes, such as *CDKN1A* (p21), are involved in cell cycle regulation leading to cell cycle arrest^15^ while others are involved in apoptosis^16^.

Previous studies investigating genome-wide IR-induced gene expression changes have found hundreds of genes responsive to IR in an ATM- and p53-dependent manner both in cell culture^17^,^18^,^19^,^20^ and *in vivo*^21^. Since these studies were based on steady-state RNA analyses, it was not possible to distinguish whether these IR-induced changes in RNA levels were due to alterations in the synthesis or turnover of RNA. In fact, studies have shown that certain transcripts may become stabilized following exposure of cells to DNA damage^22^,^23^. Thus, it is possible that regulation of RNA stability is an important strategy that cells employ in addition to regulating transcription initiation to re-program gene expression following exposure to IR.

To obtain a comprehensive picture of the transcriptional and post-transcriptional profile of the ionizing radiation response in human cells, we used the recently developed Bru-seq, BruChase-seq and BruUV-seq techniques^24^,^25^,^26^. Since these approaches are based on the labeling and isolation of nascent RNA using bromouridine (Bru), rapid changes in transcription rates can be captured without the confounding influence of pre-existing, steady-state RNA. Furthermore, by using a chase protocol after Bru labeling, relative RNA stability can be assessed genome-wide. Finally, the BruUV-seq technique allowed us to precisely map enhancer elements induced by ionizing radiation. This study shows for the first time that cells rapidly reprogram gene expression by both transcriptional and post-transcriptional mechanisms involving ATM and p53 following exposure to ionizing radiation.

## Results

### IR induced alterations of RNA synthesis in human fibroblasts

**Figure 1:**
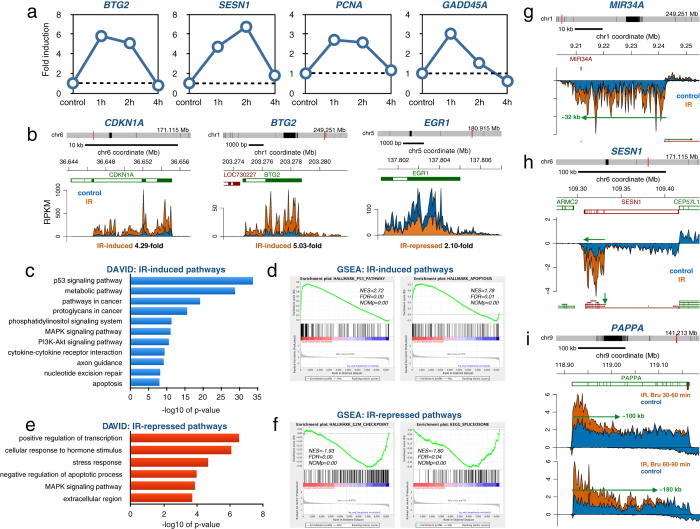
IR-induce changes in transcription in human fibroblasts (**a**) Human HF1 fibroblasts cells were mock-irradiated (0 h) or irradiated with 2 Gy and labeled for 60 min with 2 mM Bru either 1, 2 or 4 hours after irradiation. Bru-labeled RNA was immuno-captured and subjected to RT-PCR using the human DNA damage-response RT-PCR array. Time-course data is shown for *BTG2, SESN1, PCNA* and *GADD45A* transiently induced by IR. **(b)** Bru-seq data for *CDKN1A, BTG2* and *EGR1* from HF1 fibroblast cells either mock-irradiated (blue) or exposed to 2 Gy and labeled with 2 mM Bru for 30 min after a 1-hour post-incubation (orange). **(c)** DAVID functional annotation analysis of 250 genes showing significantly induced transcription following exposure to 2 Gy of IR in HF1 cells according to DESeq (n = 2). **(d)** Display of the highly IR-induced “p53 pathway” and “apoptosis” according to GSEA analysis listing the normalized enrichment score (NES), false discovery rate q-value (FDR) and the nominal p-value (NOMp). **(e)** DAVID functional annotation analysis of 33 genes showing significantly suppressed transcription following exposure to 2 Gy of IR in HF1 cells according to DESeq (n = 2). **(f)** Display of the highly IR-suppressed “G2/M checkpoint” and “spliceosome” according to GSEA analysis. **(g)** Induction of a 32 kb primary transcript of the *miR34A* gene 1 h after exposure of HF1 fibroblasts to 2 Gy. **(h)** IR-specific induction of transcription from the promoter of the short isoform but not the promoter of the long isoform of *SESN1*. **(i)** Delayed completion of IR-induced transcription of the >200 kb long *PAPPA* gene. The front of the IR-induced transcription wave had reached ~100 kb within 60 minutes and ~180 kb within 90 min after irradiation.

To explore the effect of IR on transcriptional regulation of gene expression in human cells, we chose the clinically relevant dose of 2 Gy, which is frequently used in fractionated radiotherapy for treating cancer^2^. To interrogate the kinetics of transcriptional responses following IR, we labeled HF1 human fibroblasts^27^,^28^ with 2 mM Bru for 60 min at 1, 2 or 4 hours after exposure to 2 Gy. Using RT-PCR array analysis of the captured nascent RNA it was found that transcription rates peaked within 1–2 hours after exposure for many of the induced genes (Fig. 1a). We next used Bru-seq to explore the profile of nascent RNA synthesis genome-wide in HF1 cells following irradiation with 2 Gy and performed the 30 min Bru-labeling 1 hour after irradiation, the time of peak gene expression changes. The experiments were performed in duplicate and the two experiments showed high reproducibility (Supplemental Fig. 1). It was found that 250 genes showed significantly induced transcription and 33 genes showed significantly reduced transcription after IR (padj < 0.1, mean RPKM ≥ 0.5 and gene length >300 bp), as determined using DESeq^29^ (Supplemental Table 1). Genes known to be induced following IR, such as CDKN1A (p21) and BTG2, showed 4- and 5-fold induction of transcription 60–90 min after exposure to 2 Gy, respectively, while EGR1 showed a 2.1-fold transcriptional repression (Fig. 1b). DAVID analysis^30^ on the gene list with significantly induced transcription showed as expected induction of “p53 signaling pathway” and also “metabolic pathway” and “proteoglycans in cancer” (Fig. 1c). Gene Set Enrichment Analysis (GSEA)^31^ of a pre-ranked list of all genes expressed at and above 0.5 RPKM showed enrichment of “p53 pathway” and “apoptosis” (Fig. 1d). Genes with significantly repressed transcription showed with DAVID analysis enrichment of “positive regulation of transcription”, “cellular response to hormone stimulus” and “MAPK signaling pathway”(Fig. 1e) while GSEA analysis showed enrichment for “G2/M checkpoint” and “spliceosome” (Fig. 1f).

Since primary miRNA transcripts (pri-miRNAs) are rapidly processed by DROSHA into much shorter pre-miRNAs^32^, pri-miRNAs have been difficult to annotate from studies using standard RNA-seq techniques. Analysis of nascent RNA with Bru-seq allows for the capturing of unstable long-noncoding RNA (lncRNA), such as the primary transcript of miRNA34a that is induced rapidly after exposure to IR (Fig. 1g). As can be seen, the promoter driving the expression of *MIR34a* is located ~32 kb upstream of the annotated *MIR34A* gene. Additional unstable lncRNAs that show induced transcription following IR in HF1 cells are shown in Supplemental Fig. 2. Using Bru-seq, we were also able to assess transcription originating from different promoters of multi-promoter genes. An example of a gene utilizing multiple promoters is *SESN1* where the promoter of the shorter isoform responded to IR while the promoter of the long isoform did not (Fig. 1h). Since Bru-seq captures nascent RNA, it allows for the dynamic assessment of transcription regulation of acute cellular responses^28^. To assess how quickly transcription is induced following exposure of cells to IR, we exposed HF1 cells to 2 Gy IR and labeled the nascent RNA either 30–60 min or 60–90 min after irradiation. Examination of IR-induced transcription in the very large IR-responsive gene *PAPPA* revealed that the wave of induced transcription had reached about 100 kb into the gene after the 30–60 min Bru-labeling period while it had advanced about 180 kb into the gene by the end of the 60–90 min labeling (Fig. 1i). Since the median rate of transcription elongation has been estimated to be around 1.5 kb/min in the beginning of human genes^33^,^34^,^35^ and that transcription accelerates further into the bodies of long genes^28^,^34^, the transcriptional initiation of the *PAPPA* gene must have occurred very rapidly after exposure to IR before any accumulation of p53 could have occurred. Similar elongation rates were previously obtained using GRO-seq following gene induction by estrogen^36^, TNF^36^ or serum stimulation^28^. These examples highlight the usefulness of Bru-seq in capturing unstable lncRNA transcription, determining isoform-specific transcription regulation and assessing the dynamics of induction and elongation of transcription.

### Restriction enzyme cutting and p53 induction induce similar transcriptional profile as IR

**Figure 2:**
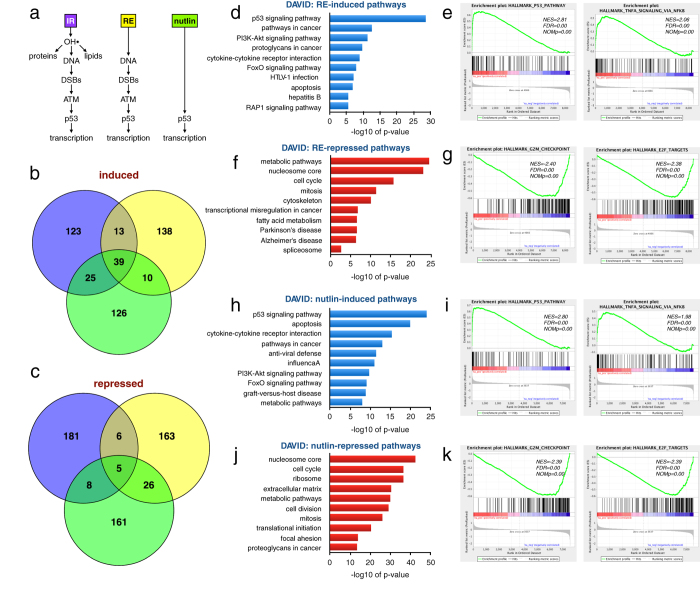
Comparing transcriptional responses to IR, restriction enzyme (RE)-induced DSBs and the p53 activator Nutlin-3. **(a)** IR induces damage to DNA, proteins and lipids while induction of the restriction enzyme AsiSI only causes DNA DSBs and Nutlin-3 induces p53 without DNA damage. **(b)** Venn diagram comparing the top 200 induced genes for the three different treatments. There were 39 genes induced by all three treatments. **(c)** Venn diagram comparing the top 200 repressed genes for the three treatments. Only 5 genes were repressed by all three of the treatments. **(d)** DAVID functional annotation analysis of genes induced >1.5-fold following 4 hours of ER-AsiSI activation by tamoxifen. **(e)** Top Hallmark pathways “p53 pathway” and “TNF signaling by NFkB” induced by RE listing the normalized enrichment score (NES), false discovery rate q-value (FDR) and the nominal p-value (NOMp). **(f)** DAVID analysis of genes suppressed >1.5-fold following 4 hours of ER-AsiSI activation by tamoxifen. **(g)** Top Hallmark pathways “G2/M checkpoint” and “E2F targets” suppressed by RE. **(h)** DAVID functional annotation analysis of genes induced >2-fold following 6 hours of nutlin-3 treatment. **(i)** Top Hallmark pathways “p53 pathway” and “TNFA signaling by NFkB” induced by nutlin-3. **(j)** DAVID analysis of genes suppressed >2-fold following 6 hours of Nutlin-3 treatment. **(k)** Top Hallmark pathways “G2/M checkpoint” and “E2F targets” suppressed by Nutlin-3.

In addition to causing DNA damage, IR inflicts damage to lipids and proteins^2^,^3^. To distinguish between DNA damage and damage to other biomolecules as triggers for reprogramming of gene expression after IR, we used Bru-seq to profile transcriptional alterations in cells in which DSBs were induced by the restriction enzyme AsiSI (RE) and in cells treated with Nutlin-3 to activate p53 without causing damage to either DNA, lipids or proteins (Fig. 2a). Comparing the top 200 genes showing induced transcription revealed 39 genes common between the three treatment groups (Fig. 2b, Supplemental Table 2), many of which were known p53 target genes (Supplemental Fig. 3). Examples of genes induced by all three groups were *CDKN1A* and the non-coding RNA genes *MIR34A* and *LINC01021* (Supplemental Fig. 4). In contrast, we only found 5 genes in common between the three groups that showed reduced transcription, including 4 histone genes and the KIF20A gene encoding a protein required for mitotic cytokinesis (Fig. 2c, Supplemental Table 2). DSBs induced by the restriction enzyme and p53 induced by Nutlin-3 resulted in transcriptional changes of genes belonging to similar groups (Fig. 2d–k) such as induction of “p53 signaling pathway”, “pathways in cancer” and inflammatory responses involving TNF signaling and repression of “mitotic cell cycle”, “G2/M checkpoint” and “E2F targets”. Thus, many of the genes induced and repressed as part of the cellular response to IR are also responding to DSBs induced by a restriction enzyme and by direct activation of p53 by Nutlin-3, suggesting that it is p53-activation in response to DSBs that drives the transcriptional re-programming after IR.

### IR causes alterations in the stability of specific RNAs

**Figure 3:**
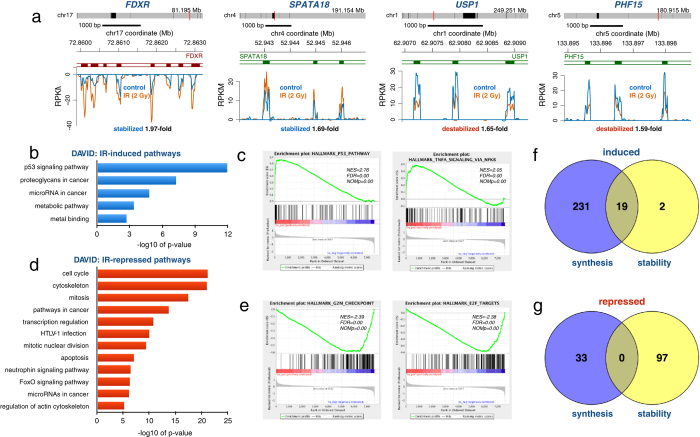
IR affects RNA stability. **(a)** The FDXR and *SPATA18* transcripts were stabilized while the *USP1* and *PHF15* transcripts were destabilized by IR as measured by BruChase-seq. HF1 cells were Bru-labeled for 30 min and then irradiated with 2 Gy (orange trace) or not (control blue trace) and chased in uridine for 6 hours. **(b)** DAVID functional annotation analysis of 21 transcripts significantly stabilized according to DESeq (n = 2). **(c)** Top Hallmark pathways “p53 pathway” and “cholesterol homeostasis” induced by IR, listing the normalized enrichment score (NES), false discovery rate q-value (FDR) and the nominal p-value (NOMp). **(d)** DAVID functional annotation analysis of 97 transcripts significantly destabilized according to DESeq (n = 2). **(e)** Top Hallmark pathways “E2F targets” and “G2/M checkpoints” of transcripts destabilized by IR. **(f)** Venn diagram showing the correlation between genes up regulated either by transcription (231), stability (2) or both (19). Data is from Supplemental Tables 1 and 5. **(g)** Venn diagram showing the correlation between genes down regulated either by transcription (33), stability (97) or both (0). Data is from Supplemental Tables 1 and 5.

The stability of RNA is a critical determinant of the steady-state level of RNA in cells. To assess whether IR-induced gene re-programming involves the regulation of mRNA stability, we performed BruChase-seq experiments on HF1 cells which were either mock-irradiated or irradiated with 2 Gy directly after a 30-min Bru-labeling. The rationale was that the control and irradiated samples would be Bru-labeled identically and only if IR affected the post-transcriptional regulation of RNA turnover would we observe any differences in the amount of Bru-RNA captured after a 6-hour chase^24^,^25^. In Fig. 3a are shown zoomed-in views of the 6-hour old *FDXR* and *SPATA18* transcripts, which were stabilized by IR, and the *USP1* and *PHF15* transcripts, which were de-stabilized. The IR-induced changes in RNA stability of specific transcripts were small and may be underestimated as the irradiations were conducted after the RNA was labeled and may therefore act “too late” to protect the RNA. We observed that 21 transcripts were significantly stabilized while 97 transcripts were significantly de-stabilized (padj < 0.1, mean RPKM ≥ 0.5, gene length >300 bp) following exposure to IR according to DESeq (N = 2) (Supplemental Table 3). Performing DAVID and GSEA analyses on the stabilized transcripts showed gene enrichment for “p53 signaling pathway”, “proteoglycans in cancer” and “microRNA in cancer” (Fig. 3b,c) while the de-stabilized transcripts were enriched in “cell cycle”, “mitosis”, “E2F targets” and “G2/M checkpoint” (Fig. 3d,e).

We next investigated whether the transcripts that were stabilized following IR also showed IR-mediated induction of nascent transcription. We found that 19 out of 21 (90%) of the significantly stabilized transcripts also showed up-regulated transcription following IR (Fig. 3f). Thus, it appears that IR-induced gene expression is a coordination of both transcriptional and post-transcriptional regulation. However, it is possible that despite chasing the Bru-labeled cells in 10-fold excess of uridine, some of the Bru taken up by the cells could continue to be incorporated to some extent during the chase period and therefore IR-induced transcription of particular genes may by default result in the erroneous interpretation of increased transcript stability. That the IR-induced changes in RNA stability observed were not just the result of continued Bru-incorporation during the chase was validated by the finding that many genes with induced transcription did not show a change in RNA stability (Fig. 3f). Furthermore, in Supplemental Fig. 5 are shown examples of three genes with significantly IR-induced Bru-labeling while showing no change in RNA stability. Thus, these findings suggest that the increased RNA stability estimated by BruChase-seq are not merely due to sustained Bru-labeling during the chase period but rather reflect increased half-lives of these transcripts. For the 97 significantly de-stabilized transcripts, we did not find any of them accompanied by IR-mediated suppression of nascent transcription (Fig. 3g). Taken together, these novel results imply that the rapid inductions of many genes in the p53-signaling pathway are regulated by both increased transcription and increased stability of their mRNA transcripts. This coordinated mechanism of both transcriptional and post-transcriptional regulation probably evolved to ensure a rapid expression of these gene products to efficiently circumvent the deleterious effects of DNA damage.

### ATM and p53 mediate transcriptional and post-transcriptional regulation after IR

**Figure 4:**
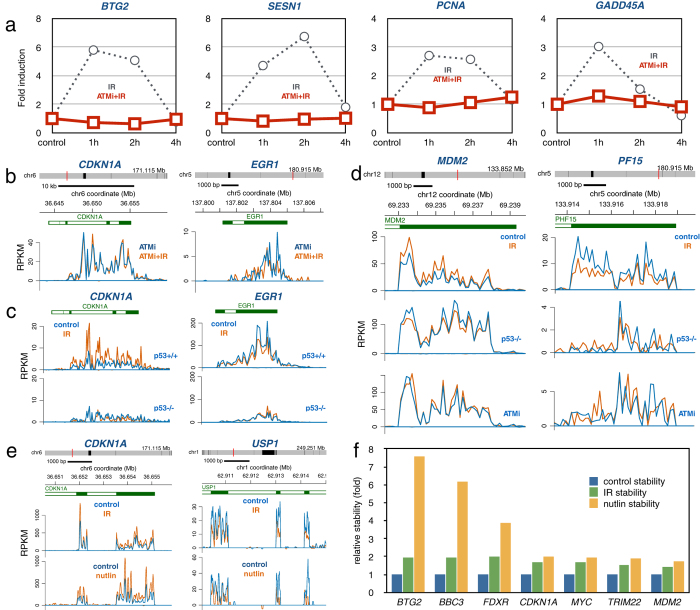
Roles for ATM and p53 in regulating both RNA synthesis and stability. **(a)** Human HF1 fibroblasts cells were pre-treated with the ATM kinase inhibitor KU55933 for 15 min and then mock-irradiated (0 h) or irradiated with 2 Gy and labeled for 60 min with 2 mM Bru 1, 2 or 4 hours after irradiation in the presence of KU55933. Bru-labeled RNA was immuno-captured and subjected to RT-PCR using the human DNA damage-response RT-PCR array. Compared to the transient transcriptional induction seen in the control samples (dashed lines, from Fig. 1a), the treatment with the ATM inhibitor abolished the IR-induced transcriptional induction of the four genes. **(b)** Bru-seq data from HF1 human fibroblasts showing that ATM inhibition with KU55933 abolishes IR induced induction of *CDKN1A* and IR-induced suppression of *EGR1*. **(c)** Bru-seq data from human colorectal cancer cells HCT116 p53+/+ and HCT116 p53−/− showing the importance of p53 in inducing transcription of the *CDKN1A* gene and suppressing transcription from the *EGR1* gene. **(d)** IR-induced stabilization of the *MDM2* transcripts and the de-stabilization of the PF15 transcript (top panels) were abolished by the absence of p53 (middle panels) and in the presence of the ATM inhibitor KU55933 (bottom panels). **(e)** The IR-induced stabilization of the *CDKN1A* transcript and de-stabilization of the *USP1* transcript was mimicked by a 6-hour treatment with Nutlin-3. (**f**) Induced stability by IR (green) or Nutlin-3 (yellow) of transcripts generated from *BTG2, BBC3, FDXR, CDKN1A, MYC, TRIM22* and *MDM2* expressed relative to untreated control HF1 cells (blue). Control and IR samples represent two independent experiments while the Nutlin-3 sample represents a single experiment.

To explore the role of the ATM kinase in the transcriptional regulation after IR, we irradiated cells in the presence of the ATM inhibitor KU55933 and performed RT-PCR array analysis at different time points. The results show that inhibition of ATM abolishes transcriptional induction of *BTG2, SESN1, PCNA* and *GADD45A* following IR (Fig. 4a). We then performed genome-wide Bru-seq analysis and similarly found that inhibition of the ATM kinase blocked IR-mediated induction and repression of transcription. The *CDKN1A* gene, which showed robust induction of transcription in response to IR, and the IR-repressed gene *EGR1*, showed no IR-mediated change in transcription when assayed in the presence of KU55933 (Fig. 4b). We next explored the contribution of p53 in the regulation of transcription following IR. We used two isogenic HCT116 colon cancer cell lines, one harboring wild-type p53 while the other lacking p53 protein expression^37^. Lack of p53 expression resulted in abrogated induction of the *CDKN1A* gene and loss of repression of the *EGR1* gene following IR (Fig. 4c). Thus, like ATM kinase activity, p53 function is required for transcriptional re-programming following IR.

Next we addressed the roles of ATM and p53 in regulating IR-induced alterations in transcript stability. IR-induced increased stability of the *MDM2* transcript was abolished in the absence of functional p53 or ATM (Fig. 4d). Similarly, IR-induced de-stabilization of the *PF15* transcript was negated by loss of p53 or ATM activity. Furthermore, induction of p53 by treatment with Nutlin-3 caused stabilization of the CDKN1A transcript and de-stabilization of the USP1 transcript similarly to IR (Fig. 4e). Enhanced transcript stability by both IR and nutlin was also observed for *MDM2, SEPT11, AURKA* and *CCNA2* (Supplemental Fig. 6) and for *BTG2, BBC3, FDXR, MYC* and *TRIM22* (Fig. 4f). Thus, these results show that, similar to gene transcription, both ATM and p53 are required for the post-transcriptional regulation of transcript stability following IR.

### IR causes activation of specific enhancer elements in an ATM and p53-mediated manner

**Figure 5:**
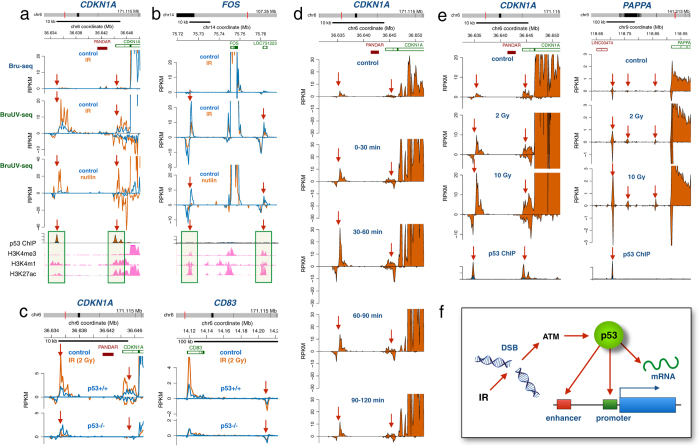
IR-induced activation and repression of enhancer elements mediated by p53. **(a)** Human HF1 fibroblasts were irradiated with 2 Gy followed by Bru-seq or BruUV-seq after 1 hour of post-IR incubation at 37 °C. Two regions upstream of the *CDKN1A* gene showed strong enhancement in reads following IR when using the BruUV-seq assay (middle panel), but not when using the Bru-seq assay (top panel). These BruUV-seq peaks also showed strong enhancement in reads following a 6-hour Nutlin-3 treatment (bottom panel). The IR- and Nutlin-3-induced BruUV-seq peaks aligned closely with p53 ChIP-seq binding data (from ref. 35) and histone modifications associated with enhancer elements such as high H3K4me1, high H3K27ac and low H3K4me3 (ENCODE data from UCSC browser) **(b)** IR-induced suppression of BruUV-seq peaks (middle panel) were also suppressed by a 6-hour Nutlin-3 treatment (bottom panel). These peaks were not associated with p53 binding peaks but showed histone modifications affirmative of enhancer elements. **(c)** The IR-induced BruUV-seq peaks upstream of the *CDKN1A* gene (left top panel) and a peak downstream of the *CD83* gene (right top panels) found in p53+/+ HCT116 cells were abolished in HCT116 cells lacking p53 (bottom panels). **(d)** BruUV-seq analyses were performed on HF1 human fibroblasts at different times after irradiation with 2 Gy and show a transient IR-mediated induction of nascent eRNA generated from the two putative enhancer elements upstream of the *CDKN1*A gene with a maximum of expression 30–60 minutes after irradiation. **(e)** IR induces eRNA associated with the *CDKN1A* and *PAPPA* genes in a dose-dependent manner in human HF1 cells. (**f**) Model of the roles of p53 in regulating the transcriptional and post-transcriptional responses following IR.

Activated p53 proteins have been shown to bind to both promoter and enhancer elements^38^,^39^,^40^,^41^,^42^,^43^,^44^. To explore whether induction of p53 by IR results in the activation of enhancer elements near genes with IR-induced transcription, we used our recently developed BruUV-seq technique^26^. This technique utilizes ultraviolet (UV) light to induce transcription-blocking DNA lesions and to inhibit the RNA exosome resulting in increased reads of eRNA generated by active enhancer elements^26^,^45^,^46^. Using BruUV-seq on HF1 cells, we found two peaks upstream of the CDKN1A gene, which were strongly enhanced by IR but not present in the Bru-seq data (Fig. 5a). These Bru UV-seq peaks were also strongly induced by Nutlin-3 suggesting that they were induced in a p53-dependent manner. Indeed, previously published p53 ChIP-seq data^38^ performed on human fibroblasts showed that Nutlin-3-activated p53 binds to these enhancer elements. Similarly, p53 co-localized with activated enhancer elements near the IR-induced genes *MAMDC2, GDF15* and *GDNF* (Supplemental Fig. 7). The reduced transcription of the *FOS* gene following IR was accompanied by reduced generation of RNA from two nearby putative enhancer elements (Fig. 5b). Similar reduction was observed following Nutlin-3 treatment but interestingly, no p53 binding was evident at these sites. Similar findings were obtained for putative enhancer elements near the IR-repressed genes KRTAP-5, FOSB and HSPA2 (Supplemental Fig. 8). In p53 null cells, IR-induced activation of the enhancer elements near the *CDKN1A* and *CD83* genes was not observed (Fig. 5c). We next explored the kinetics of enhancer activation by IR and found that generation of eRNA from the two enhancer elements upstream of the CDKN1A reached a maximum 30–60 minutes after IR, after which enhancer activity diminished (Fig. 5d). Finally, the generation of eRNA following IR was found to be dose dependent as shown for CDKN1A and PAPPA (Fig. 5e). Together, these results suggest that IR induces target genes by activating nearby enhancer elements in a time and dose-dependent manner via the binding of p53. In contrast, genes repressed by IR via p53-dependent reduced enhancer activity are repressed without associated p53 enhancer binding.

## Discussion

The ability of image-guided radiation therapy to deliver targeted ionizing radiation to tumors makes this therapeutic modality very attractive^47^. However, toxicity to normal tissues is dose limiting underscoring a need to better understand the biological effects of ionizing radiation to normal cells. Previous studies have shown that ionizing radiation elicits a DNA damage response in human cells primarily driven by the stress kinase ATM and the transcription factor p53 resulting in expression changes in a select set of genes^20^. While these gene expression changes were found associated with specific histone modifications suggestive of transcriptional re-programming, a distinction between changes in synthesis and turnover of RNA could not be determined from these steady-state RNA-seq studies. To explore the individual contributions of synthesis and turnover to the radiation-induced gene expression re-programming in normal human fibroblasts, we here performed genome-wide analyses of transcription and RNA stability using Bru-seq and BruChase-seq^24^,^25^, respectively. Furthermore, we explored enhancer element utilization following exposure to IR using BruUV-seq^26^.

Since assessments of transcription rates by Bru-seq are not confounded by the presence of pre-existing RNA, rapid changes in transcription can be detected. We found that 2 Gy of ionizing radiation induced a transient induction of transcription in normal human fibroblasts with a peak at about 1 hour after exposure (Fig. 1). The induction and repression of transcription by ionizing radiation was primarily triggered by DSBs and mediated by p53 since similar transcription patterns were induced by DSBs induced by a restriction enzyme and by direct activation of p53 with Nutlin-3 (Fig. 2). One of our most interesting an novel findings was that ionizing radiation regulated gene expression post-transcriptionally (Fig. 3). The changes recorded for RNA stability were smaller compared to changes observed for transcription. However, since we exposed cells to IR after they had incorporated Bru into the nascent RNA to be measured, it is likely that sufficient time would have passed during which the transcripts would be expected to be targeted by the degradation machinery before the DDR-mediated actions regulating RNA stability could take effect. Thus, the amplitude of these changes may be underestimated due to our experimental set-up.

An important and novel finding of our study was that the transcriptional and post-transcriptional changes in gene expression induced by IR were dependent on both ATM signaling and p53 activity (Fig. 4). It is known that activated ATM kinase phosphorylates a large number of proteins involved in mRNA post-transcriptional regulation^8^. Following DNA damage, the *CDKN1A* transcript is stabilized by the RNA-binding protein HuR which is indirectly regulated by ATM via the CHK1 and CHK2 kinases^48^. However, the requirement of p53 for the IR-induced stabilization of the CDKN1A mRNA is not explained by activation of HuR since this protein is not known to be regulated by p53^49^. A number of p53 target genes, such as *ZMAT3*^50^,^51^, *RBM24*^52^ and *RBM38*^53^ encode RNA-binding proteins that regulate mRNA stability. While the *ZMAT3* (WIG-1) gene was transcriptionally induced in this study in the human fibroblasts after IR (~2-fold), some of its target genes, such as *TP53* and *FAS,* did not show significant stability changes of their mRNAs. Furthermore, the putative p53 target genes *RBM24* and *RBM38* were not significantly induced transcriptionally in the human fibroblasts after IR. Taken together, while this study shows requirements of ATM and p53 in the post-transcriptional regulation of gene expression following IR, the true mechanism by which they regulate transcript stability is yet to be elucidated.

In conclusion, we show that radiation induces rapid changes in the synthesis of many RNAs and that many of these transcription changes were linked to alteration in the activity of nearby enhancer elements. Additionally, many transcripts were regulated post-transcriptionally adding a new dimension to the regulation of the DNA damage response. Importantly, both ATM and p53 were required for all aspects of the transcriptional and post-transcriptional regulation of the cellular radiation response (Fig. 5e). Whether the role of p53 in regulating RNA stability is direct^54^ or indirect via induction of RNA-binding proteins such as WIG-1^51^ has to be clarified. In addition to regulating both synthesis and stability of select transcripts after IR, activation and repression of enhancer elements was found to be p53-dependent (Fig. 5). Interestingly, activation of enhancer elements correlated with induced binding of p53 to these elements while no p53 binding was found at enhancer elements suppressed by p53. Future studies will aim at elucidating how cancer-associated mutations affect these different roles of p53 in transcriptional and post-transcriptional regulation of the radiation response.

## Methods

### Cell cultures

hTERT immortalized human diploid foreskin fibroblasts (HF1) (a gift from Dr. Mary Davis, Department of Radiation Oncology, University of Michigan, Ann Arbor) were grown in MEM growth media (Minimal Essential Medium, 10% FBS, 1× MEM Amino Acids (ThermoFisher Scientific), 1× Non-Essential Amino Acids (ThermoFisher Scientific), 2 mM l-glutamine, 1X antibiotic–antimycotic (Invitrogen), 1× MEM vitamin mixture, 0.15% (w/v) sodium bicarbonate^24^,^25^. HCT116 p53+/+ and HCT116 p53−/− (Clone 379.2) (a generous gift from Dr. Bert Vogelstein, Johns Hopkins Cancer Center)^37^,^55^ used in this study were grown using RPMI 1640 (Gibco, ThermoFisher Scientific) supplemented with 10% FBS and 1% Penicillin/Streptomycin. U2OS human bone osteosarcoma cells (ATCC) were grown in McCoy’s 5a Medium Modified (ATCC) supplemented with 10% FBS and 1% Penicillin/Streptomycin.

### Bru-Seq and BruChase-Seq

For the Bru-seq experiments, cells were incubated in media containing bromouridine (Bru) (Aldrich) at a final concentration of 2 mM for 30 minutes at 37 °C to label nascent RNA. Following Bru-labeling, cells were lysed directly in Trizol followed by isolation of total RNA, immunocapturing of Bru-labeled RNA using anti-BrdU antibodies, preparation of strand-specific DNA libraries with the Illumina TruSeq Kit (Illumina) and deep sequencing using the Illumina sequencing platform as previously described^24^,^25^. For BruChase-Seq experiments, cells were first labeled with 2 mM Bru for 30 minutes, washed in PBS, conditioned media containing 20 mM uridine was added, and the cells were then either irradiated or nutlin-3 was added during the “chase” for 6 hours. The cells were lysed in Trizol and Bru-labeled RNA was captured and processed as described above. Since the control and treated samples are identically labeled, stability could be assessed by direct comparisons of the 6 h data for two IR samples and one control sample.

### BruUV-Seq

To map enhancer elements responding to IR we used the newly developed BruUV-seq technique. Here UV light is utilized to enhance the reads over enhancer elements by stabilizing eRNA through the inactivation of the nuclear RNA exosome^26^. The cells were irradiated with 20 J/m^2^ UV light at different times following exposure to IR or nutlin-3. The irradiation source (Philips, New York, NY) generated UVC light with a dose rate of 1 J/m^2^/s which was measured with a UVX radiometer (UVP, Inc. Upland CA)^8^. Following UV-irradiation, the cells were immediately labeled with 2 mM Bru for 30 min at 37 °C and processed as previously described^26^.

### Cell treatments

Ionizing radiation was administered to the HF1 and HCT116 cells using an IC-320 Biological Irradiator (Kimtron Inc., Oxford, CT) with a dose rate of ~2 Gy/min of x-rays. The MDM2 antagonist Nutlin-3 (Aldrich) was used at a concentration of 10 µM. The ATM kinase inhibitor KU55933 (A gift from Dr. Diane Simeone, University of Michigan, Ann Arbor) was added at a concentration of 10 μM 15 minutes prior to IR and remained in the media throughout the post-incubation and Bru-labeling period. For the control samples, the HF1 cells were treated with KU55933 for 1 h 15 min, after which they were Bru labeled for 30 minutes in the presence of KU55933. For the BruChase-Seq experiment, the HF1 and HCT116 cells were first Bru labeled then exposed to either IR or Nutlin-3 and a 6-hour incubation in conditioned media containing 20 mM uridine and for the Nutlin-3 samples in the presence of Nutlin-3.

### DSBs induced by Restriction Enzyme (RE)

The ER-AsiSI inducible system was used to induce about 200 DSBs directly in the genome of U2OS cells^56^,^57^. The RE was induced by addition of tamoxifen (TAM) to the cell media and the nascent RNA was labeled 210–240 min after addition of TAM. ER-U2OS cells not exposed to TAM were used as controls.

### RT-PCR arrays

HF1 fibroblasts were irradiated with 2 Gy and incubated in Bru-containing media for 60 min at different times after irradiation. The Human DNA Damage Response RT-PCR array (Catalogue no. PAHS-029, SABiosciences, now Qiagen) was used. Bru-RNA was isolated by our laboratory, and the subsequent analyses were performed by the DNA Sequencing Core, University of Michigan, Ann Arbor.

### Bru-seq data analysis

Sequenced reads were strand-specific, single-ended and 52 bp in length. RNA-seq data were aligned to the human ribosomal DNA complete repeating unit (U13369.1) using Bowtie (v0.12.8) and the reads that remained unaligned were mapped to the human genome build hg19/GRCh37 using TopHat (v1.4.1). Bru-seq and BruChase-seq data from treated samples were compared to control samples and fold differences were determined by performing DESeq (version 1.26) in R statistical software (version 3.3.1). Genes having a mean RPKM ≥ 0.5, gene length >300 bp and having a false discovery rate <0.1 (Figs 1 and 3) or a 1.5- or 2-fold expression difference (Fig. 2) were chosen for our downstream bioinformatics analyses. For the BruChase-seq data, exonic read counts at 6 hours were used to determine the fold change between the treated and the control samples. Gene set/functional enrichment analyses were performed using DAVID Functional Annotation Tools (david.abcc.ncifcrf.gov) and Gene Set Enrichment Analysis (GSEA)^31^. The gene set groups used in the GSEA analysis, Hallmark Pathways and C2: Curated gene sets (CP: KEGG gene set), were obtained from the MSigDB (Molecular Signatures Databases, v5.1, http://softwarebroadinstitute.org/gsea/msigdb/index.jsp). The gene list used for this analysis was pre-ranked by log2 fold change and included all the genes that had a mean RPKM ≥0.5 and a gene length >300 bp.

## Additional Information

**How to cite this article**: Venkata Narayanan, I. *et al*. Transcriptional and post-transcriptional regulation of the ionizing radiation response by ATM and p53. *Sci. Rep.* **7**, 43598; doi: 10.1038/srep43598 (2017).

**Publisher's note:** Springer Nature remains neutral with regard to jurisdictional claims in published maps and institutional affiliations.

## Supplementary Material

Supplemental Information

Supplemental Table 1

Supplemental Table 2

Supplemental Table 3

Supplemental Table 4

Supplemental Table 5
