# Supplementary material for: Transcriptional and post-transcriptional regulation of the ionizing radiation response by ATM and p53
Source: Sci Rep. 2017 Mar 3;7:43598. doi: 10.1038/srep43598 (PMC5335570; doi:10.1038/srep43598)
Supplement: Supplemental Information [file srep43598-s1.pdf]

SUPPLEMENTARY INFORMATION

Transcriptional and post-transcriptional regulation of the ionizing radiation response by ATM and p53

Ishwarya Venkata Narayanan, Michelle T. Paulsen, Karan Bedi, Nathan Berg, Emily A. Ljungman, Sofia Francia, Artur Veloso, Brian Magnuson, Fabrizio d'Adda di Fagagna, Thomas E. Wilson and Mats Ljungman

Table of Content:

Supplementary Figure 1: Correlation analyses for Bru-seq and BruChase-seq of irradiated HF1 cells.

Supplementary Figure 2: Bru-seq and BruChase-seq showing IR-induced transcription and stability of 5 different lncRNAs.

Supplementary Figure 3: DAVID analyses of the 39 genes commonly induced transcriptionally by IR, restriction enzyme (RE) and nutlin.

Supplementary Figure 4: Transcriptional induction of *CDKN1A*, *MIR34a* and *LINC01021* by IR, RE and nutlin.

Supplementary Figure 5: Bru-seq and BruChase-seq analysis showing increased synthesis but unchanged mRNA stability.

Supplementary Figure 6: BruChase-seq analysis showing Increased stability of transcripts generated from the *MDM2* and *SEPT11* genes and decreased stability for transcripts from the *AURKA* and *CCNA2* genes by both IR and nutlin in human HF1 fibroblasts.

Supplementary Figure 7: IR- and nutlin-induced generation of eRNA coincide with p53 binding near IR-induced genes.

Supplementary Figure 8: IR- and nutlin-suppressed generation of eRNA does not coincide with p53 binding near IR-suppressed genes.

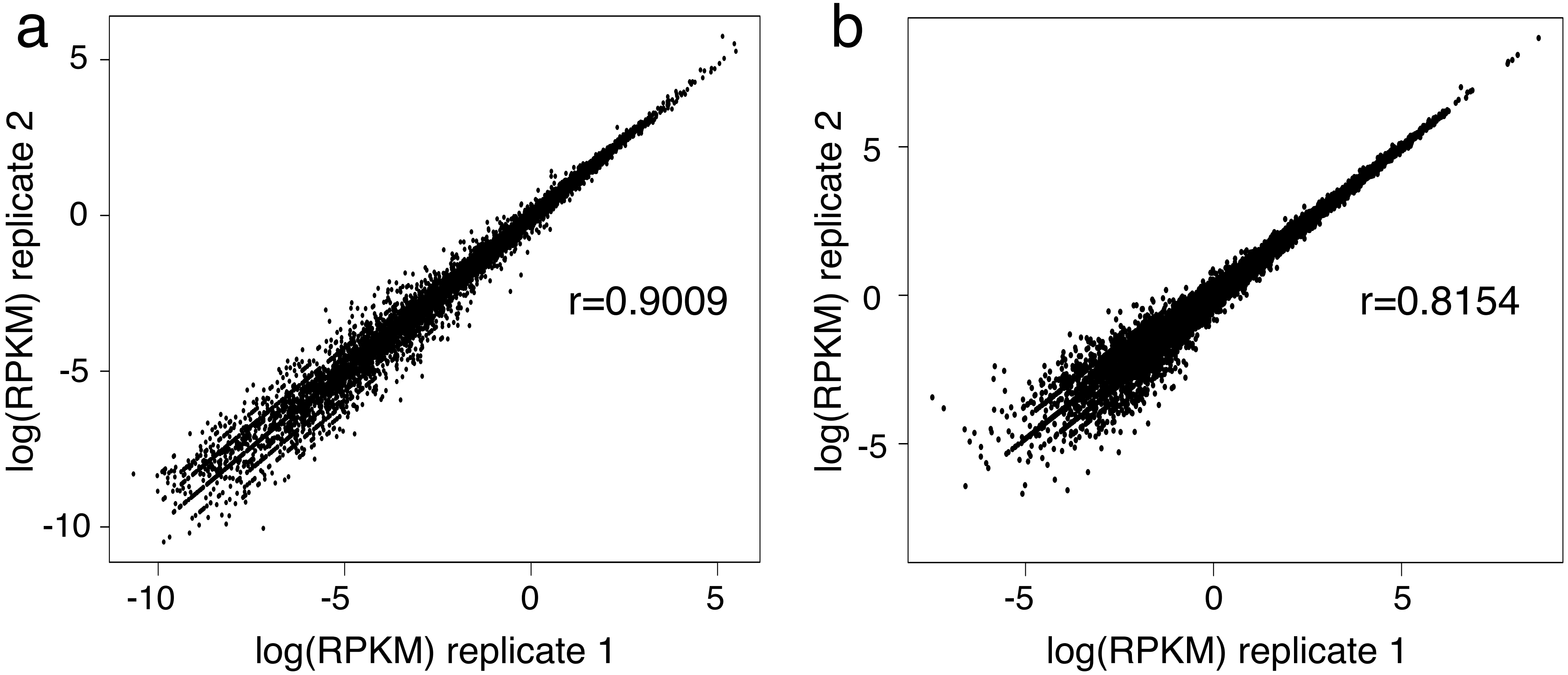

Supplemental Figure 1. Correlation analyses between two biological samples of irradiated HF1 cells for (**a**) Bru-seq (0h) and (**b**) BruChase (6h). For Bru-seq, HF1 cells were irradiated at 37°C with 2 Gy and then incubated for 60 min before Bru was added. RPKM values for the entire gene length were used for the correlation analysis. For BruChase-seq, HF1 cells were labeled for 30 min with Bru after which the cells were irradiated with 2 Gy and chased for 6 hours in media containing uridine. RPKM values for exons were used for the correlation analysis.

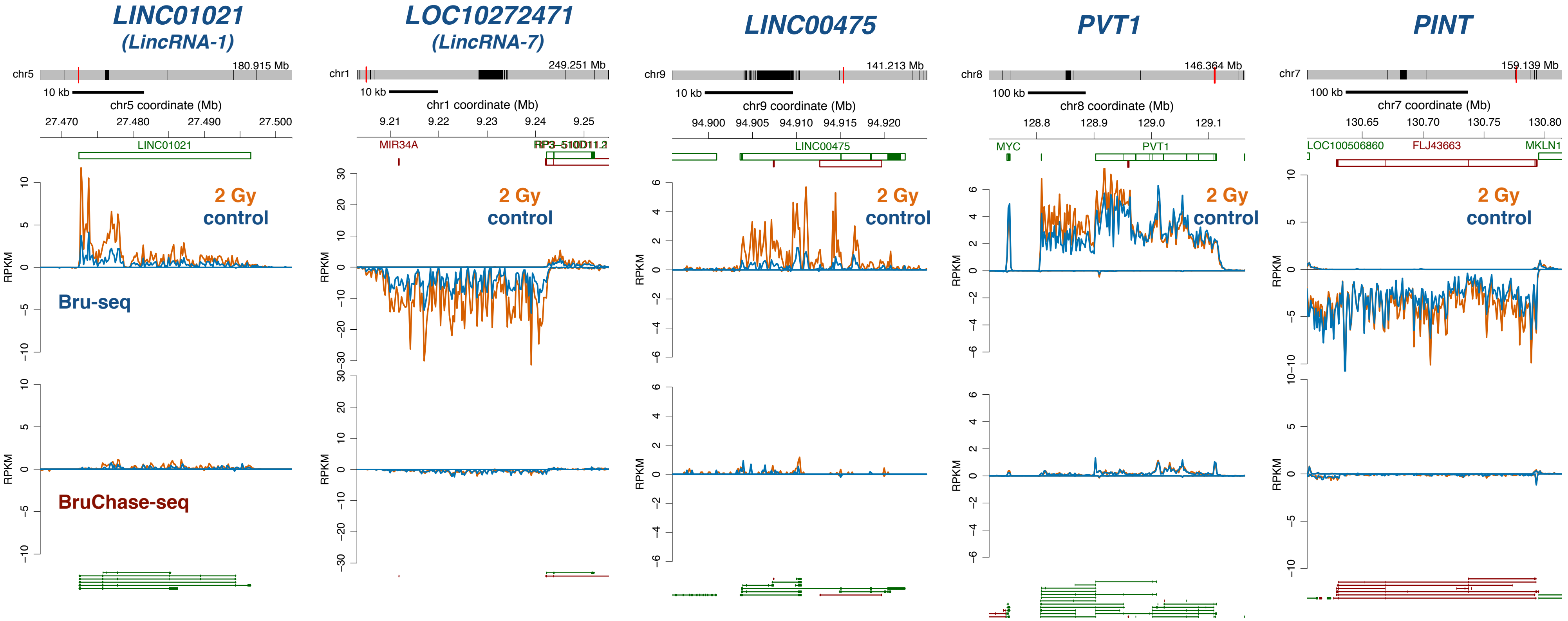

Supplemental Figure 2. Top: Bru-seq showing IR-induced transcription of 5 different lncRNAs in human HF1 fibroblasts. Bottom: BruChase-seq showing that all of these lncRNAs are unstable since very little Bru-labeled RNA were detected for these transcripts after a 6 h chase whether the cells were irradiated or not.

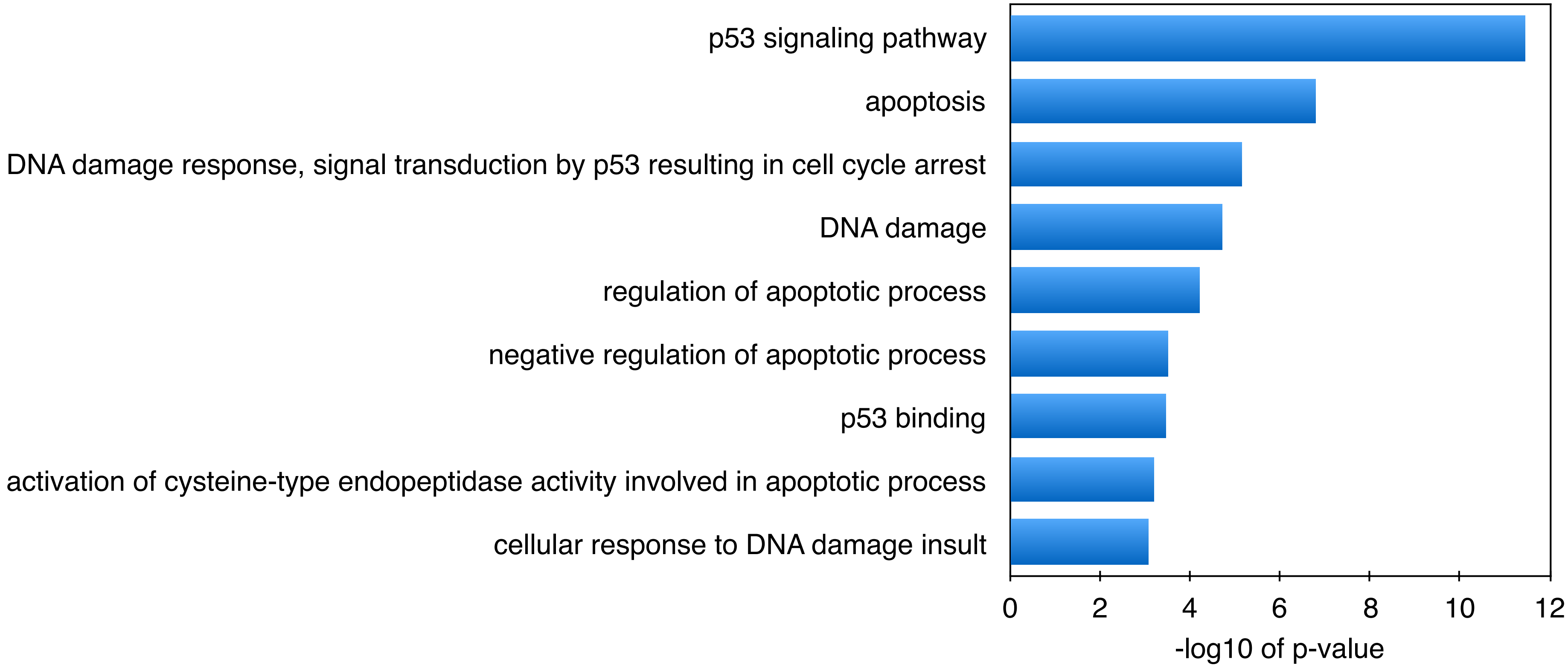

Supplemental Figure 3. DAVID analyses of the 39 genes commonly induced transcriptionally by IR, restriction enzyme (RE) and nutlin (from Figure 2b). Homo sapiens was used as background for the DAVID analysis.

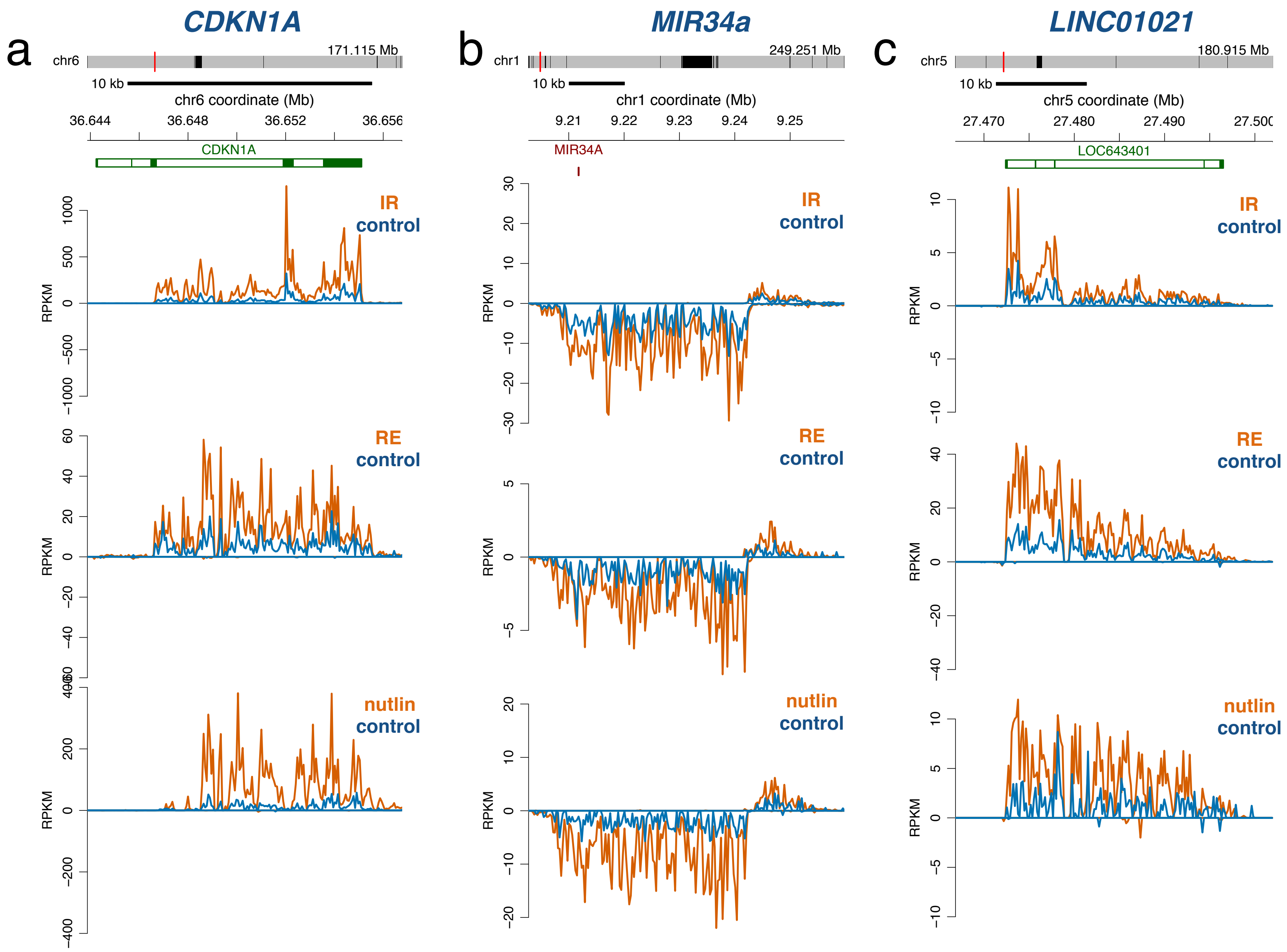

Supplemental Figure 4. Transcriptional induction of (**a**) *CDKN1A*, (**b**) *MIR34a* and (**c**) *LINC01021* by IR 1 h (top), RE(restriction enzyme AsiSI) 210 min (middle) and nutlin-3 6 h (bottom). Top and bottom samples are human fibroblasts HF1 while the middle samples are U2OS cells.

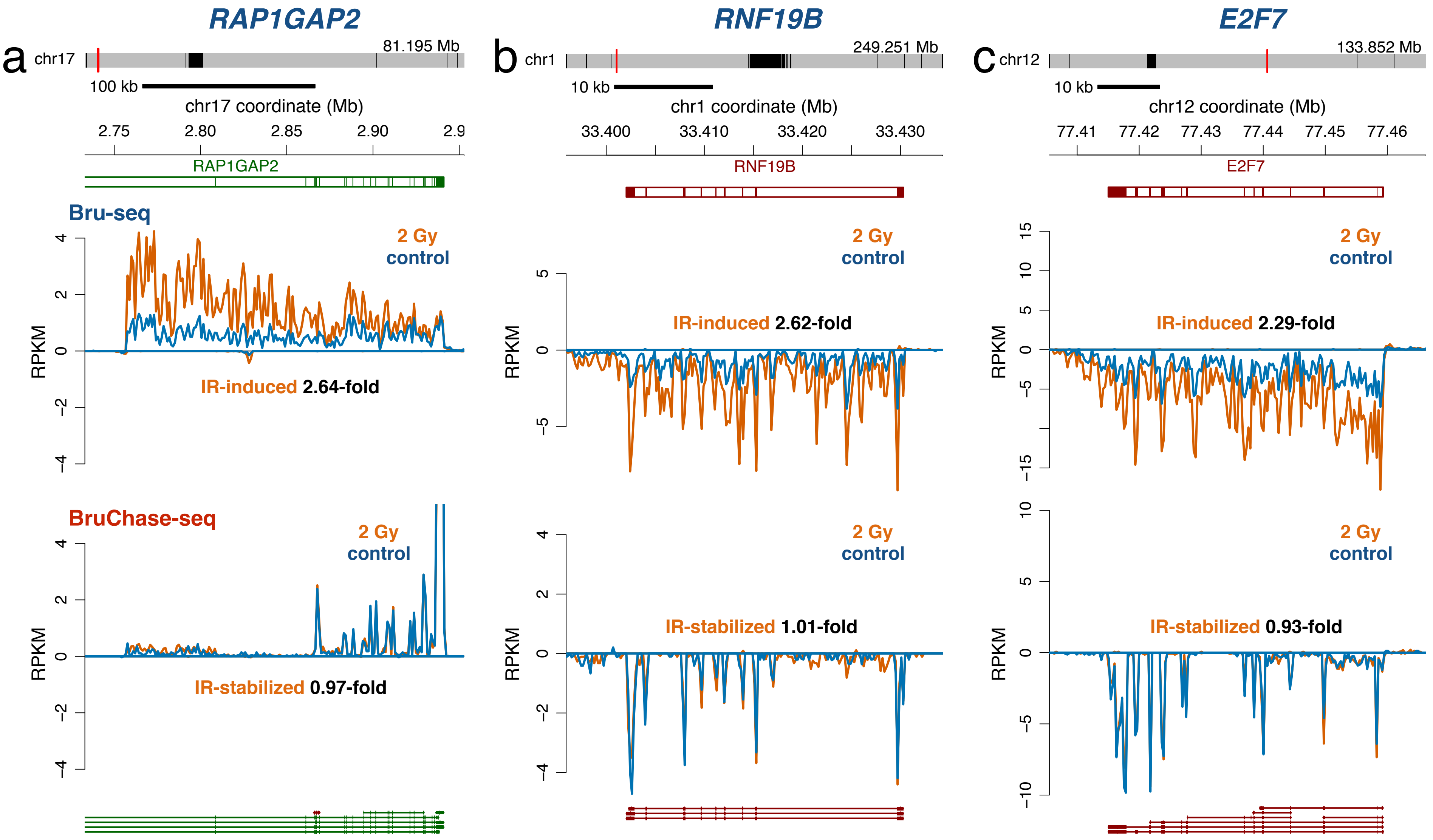

Supplemental Figure 5. Bru-seq (top) and BruChase-seq analysis (bottom) showing increased synthesis but unchanged mRNA stability of transcripts of the (**a**) *RAP1GAP2*, (**b**) *RNF19B* and (**c**) *E2F7* genes.

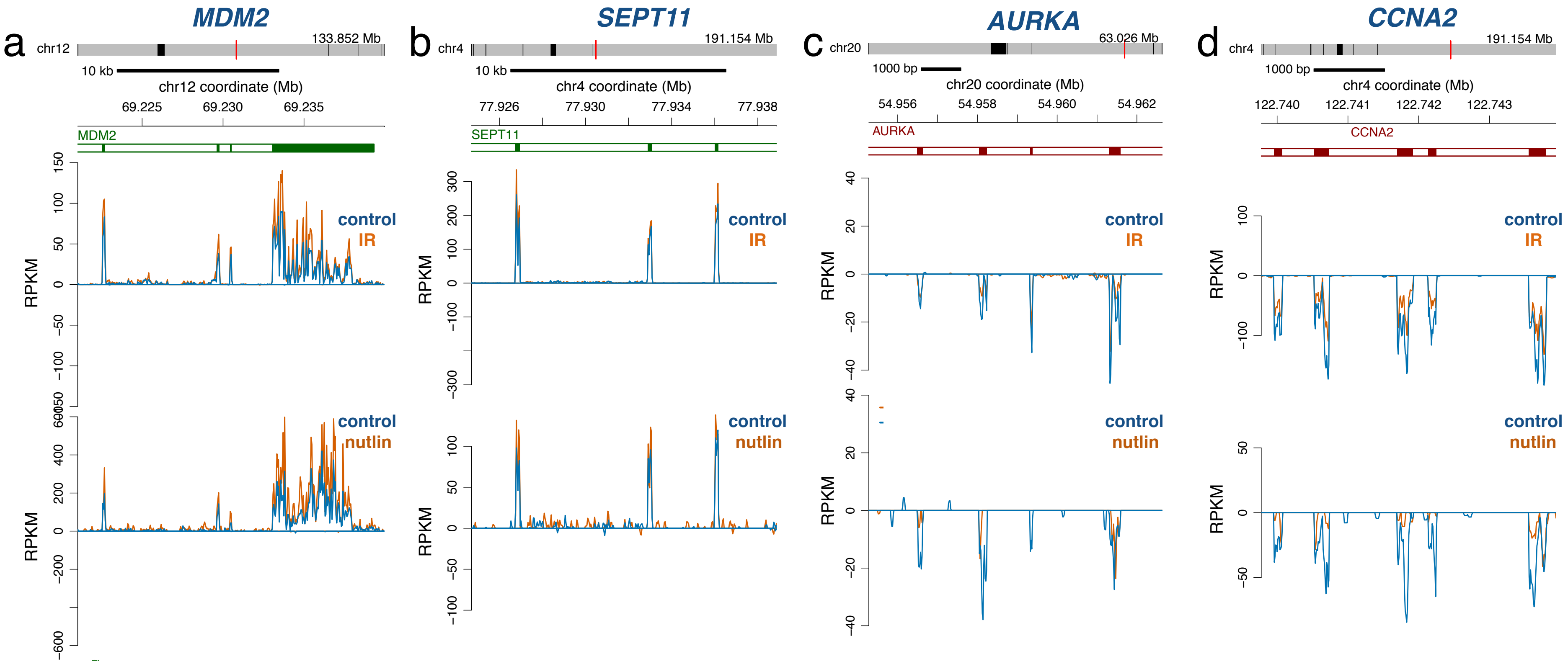

Supplemental Figure 6. BruChase-seq analysis showing Increased stability of transcripts generated from the (**a**) *MDM2* and (**b**) *SEPT11* genes and decreased stability for transcripts from the (**c**) *AURKA* and (**d**) *CCNA2* genes by both IR and nutlin in human HF1 fibroblasts. Cells were Bru-labeled for 30 min, then either exposed to IR right after Bru-labeling and before the 6 h chase (top) or nutlin-3 treatment after the Bru-labeling and during the 6 h chase period(bottom).

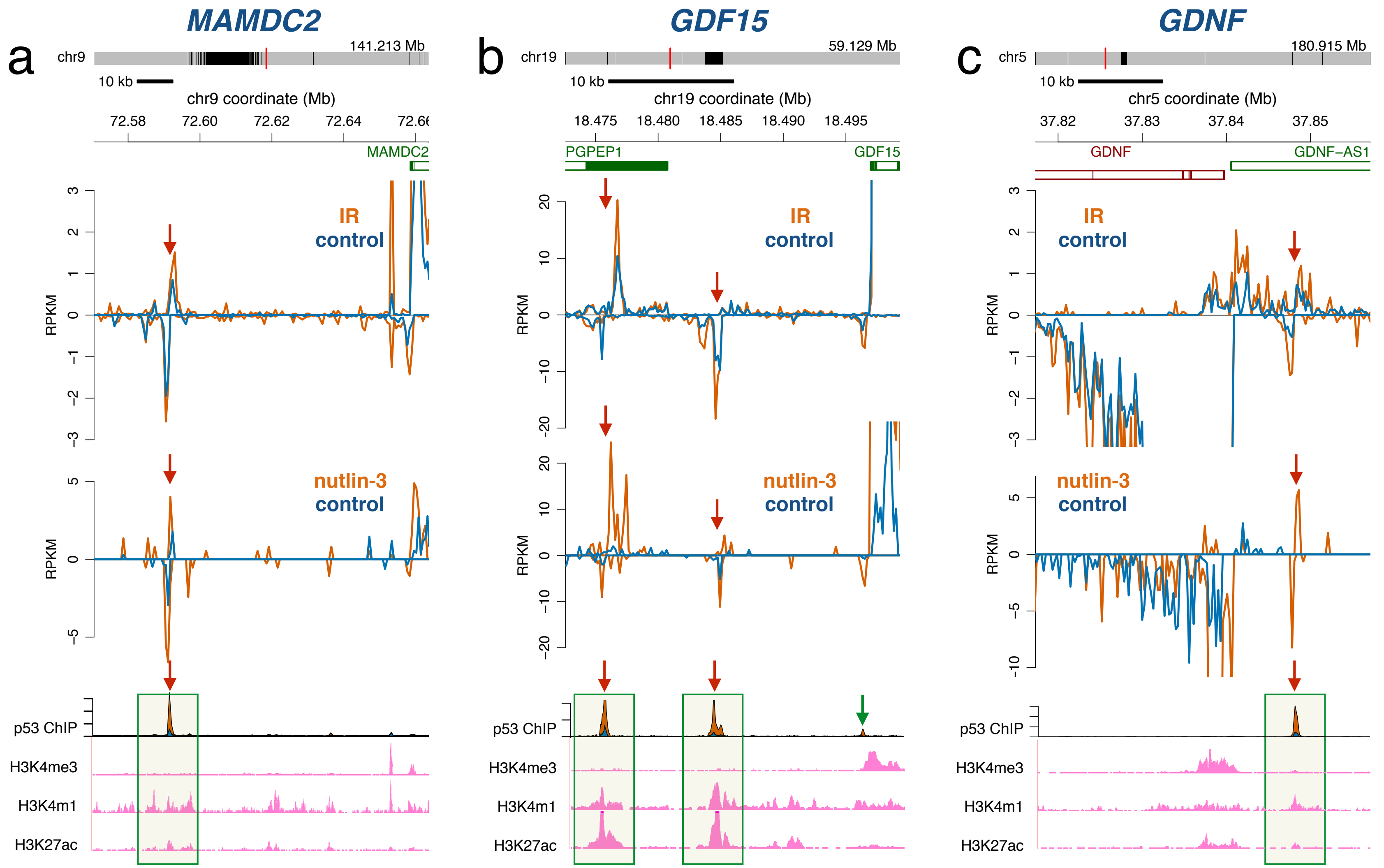

Supplemental Figure 7. IR- and nutlin-induced generation of eRNA coincide with p53 binding near IR-induced genes. (**a**) BruUV-seq showing one upstream putative enhancer elements induced following IR (top) and nutlin (middle) for the *MAMDC2* gene coinciding with a strong p53 binding site, low H3K4me3, high H3K4me1 and high H3K27ac marks (bottom). (**b**) Two upstream putative enhancer elements were induced by IR and nutlin for the *GDF15* gene. Also, one p53 binding site found at promoter (green arrow) (**c**) One upstream putative enhancer element was induced by IR and nutlin for the *GDNF* gene.

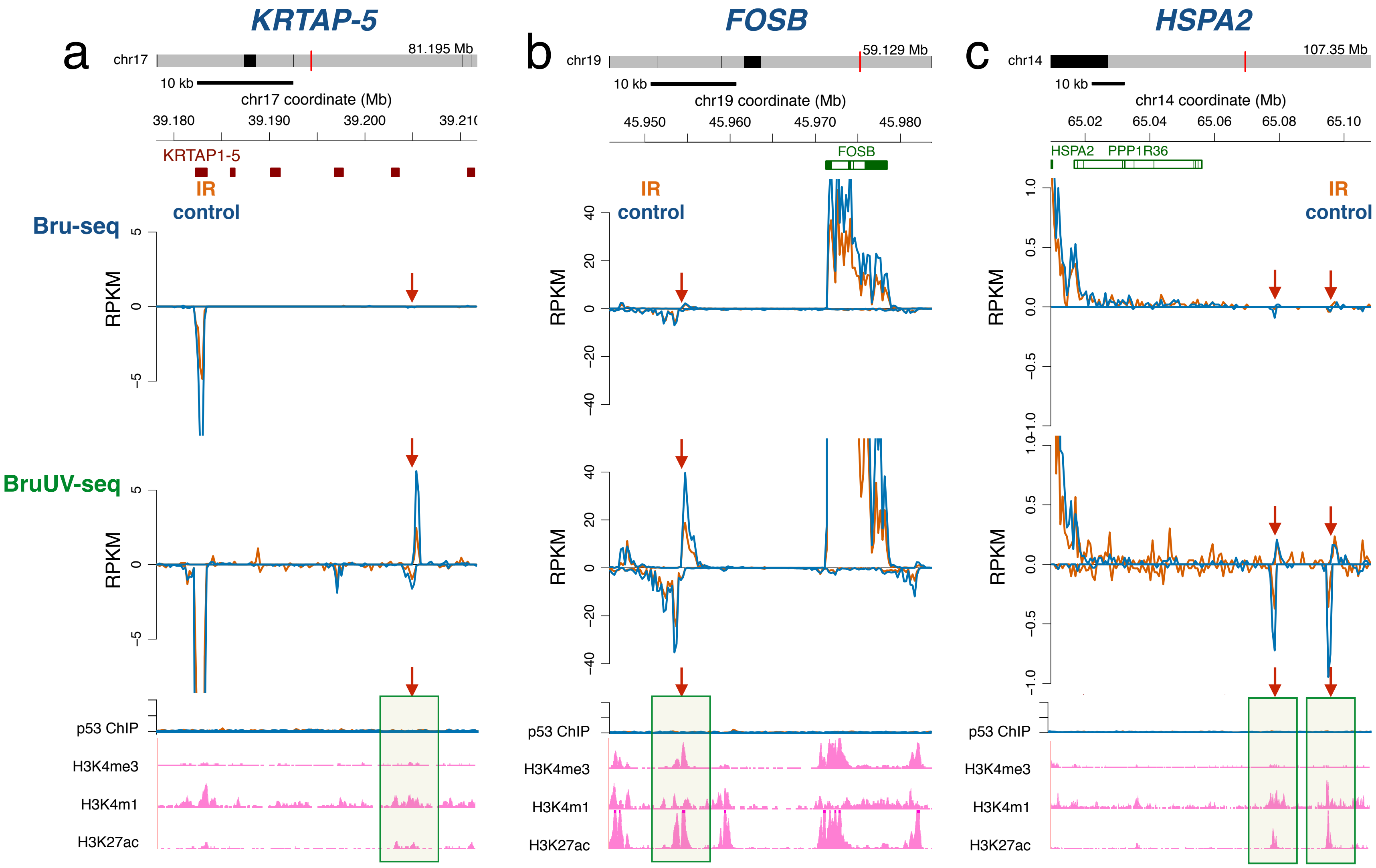

Supplemental Figure 8. IR- and nutlin-suppressed generation of eRNA does not coincide with p53 binding near IR-suppressed genes. Top: Bru-seq showing nascent transcription Middle: BruUV-seq showing IR-repressed eRNA near repressed genes. Upstream putative enhancer elements were repressed following IR (2 Gy, 1 h) for (**a**) *KRATAP-5,* (**b**) *FOSB* and (**c**) *HSPA2*. These enhancer elements were also strongly suppressed by nutlin (data not shown) but no evidence for p53-binding to these enhancer elements.
